# Genome Sequence of a Newly Isolated Nicotine-Degrading Bacterium, *Ochrobactrum* sp. SJY1

**DOI:** 10.1128/genomeA.00720-14

**Published:** 2014-07-24

**Authors:** Hao Yu, Yangyang Li, Hongzhi Tang, Ping Xu

**Affiliations:** State Key Laboratory of Microbial Metabolism and School of Life Sciences & Biotechnology, Shanghai Jiao Tong University, Shanghai, China

## Abstract

*Ochrobactrum* sp. SJY1 uses nicotine as the sources of carbon, nitrogen, and energy. The genome of SJY1 was sequenced in order to provide insights into its mechanism of nicotine degradation. Physiological characteristics and genome analysis indicate that strain SJY1 might have a different nicotine degradation pathway from the pyridine or pyrrolidine pathway.

## GENOME ANNOUNCEMENT

Degradation of nicotine by microorganisms is useful in bioremediation as it provides an environmentally friendly means of converting the toxic compound to carbon dioxide and water. Many bacteria that utilize nicotine as a carbon and nitrogen source have been isolated and characterized (1). Genetic and biochemical studies of nicotine degradation were greatly promoted by microbial genome sequencing in the past and recently (1, 2). Three genomes of nicotine-degrading strains have been published to date (3–5), including *Pseudomonas putida* S16 with the pyrrolidine degradation pathway, *Arthrobacter* sp. M2012083 with the pyridine pathway, and *Pseudomonas geniculata* N1. Recently, a Gram-negative strain, *Ochrobactrum* sp. SJY1, which uses nicotine as the sole source of carbon, nitrogen, and energy, was newly isolated from water. Strain SJY1 was deposited at the China Center for Type Culture Collection under accession number CCTCC AB 2014146. The previously isolated *Ochrobactrum bacterium*, *O. intermedium* DN2, was reported to have the capability to degrade nicotine (6) and may follow the same pyridine degradation pathway as *Arthrobacter nicotinovorans* (7). However, strain SJY1 produces no blue pigment during cultivation with nicotine, suggesting that it has a different degradation pathway from *O. intermedium* DN2. The genome sequence of *Ochrobactrum* sp. SJY1 could improve our understanding of the strain's high ability and genetic information for biodegradation of nicotine. Here we present a summary, classification, and set of features for *Ochrobactrum* sp. strain SJY1 together with a description of the genomic sequencing and annotation.

The genome of strain SJY1 was sequenced by an Illumina HiSeq-2000 sequencer (2- × 101-bp paired end). The total amount of read data comprises 1,374,835,836 bases. The reads were assembled *de novo* into 198 contigs of >500 bp (*N*_50_ contig size, 107,455 bp) using Velvet 1.2.10 software (8). The genome was functionally annotated using the Rapid Annotations using Subsystems Technology (RAST) annotation server (9). The tRNAs were predicted using tRNAscan-SE (10). The genome sequence of strain SJY1 comprises 5,245,769 bp. A total of 5,258 candidate protein-coding sequences (CDSs) were predicted, giving a coding intensity of 85.8%. The 48 tRNA genes were identified in the genome.

Thirty-six CDSs for denitrification were annotated, indicating strain SJY1 may come from anaerobic conditions. Several genes (*moaACDE*, *mobAB*, *modABC*, *moeAB*, and *mog*) for the synthesis of molybdenum cofactors and the genes from downstream of the pyrrolidine pathway (*nfo*, *ami*, and *iso*) were found in the genome of strain SJY1 (2). However, other genes, especially those upstream of the pyridine and pyrrolidine pathways, were not found in genome of strain SJY1. Sequence identity between isozymes can be observed between different strains possessing the same nicotine degradation pathway, such as strain S16 and *Pseudomonas* sp. HZN6 (2, 11, 12). These results suggests that strain SJY1 may have a different degradation pathway from the pyridine or pyrrolidine pathway. Therefore, the genome information of strain SJY1 reported here will provide new information for the study of mechanisms in nicotine degradation.

### Nucleotide sequence accession numbers.

This whole-genome shotgun project has been deposited at DDBJ/EMBL/GenBank under the accession number AZRT00000000. The version described in this paper is version AZRT01000000.
